# Efficacy and validation of a clinical predictive model for chronic atrophic gastritis in patients: a multi-center retrospective analysis

**DOI:** 10.3389/fmed.2025.1570893

**Published:** 2025-06-19

**Authors:** Xiang Fang, Wenjing Ding, Xiaolong Xu, Hui Chen, Bei Pei, Yi Zhang, Biao Song, Xuejun Li, Li Yao

**Affiliations:** ^1^Intensive Care Unit, Hefei Second People’s Hospital, Hefei, China; ^2^The Second Clinical Medical School, Anhui University of Chinese Medicine, Hefei, China; ^3^Department of Gastroenterology, The Second Affiliated Hospital of Anhui University of Chinese Medicine, Hefei, China

**Keywords:** chronic atrophic gastritis, risk factors, predictive model, nomogram, patient

## Abstract

**Background:**

Chronic atrophic gastritis (CAG) is a major digestive disorders, and prognosis is determined by many social-demographic and clinicopathologic characteristics. This study aimed to identify risk factors and construct a predictive model for better diagnosis of CAG.

**Methods:**

We utilized a multi-center retrospective analysis, including 539 cases of CAG patients diagnosed and treated in Second Affiliated Hospital of Anhui University of Chinese Medicine from September 2018 to December 2024 as training dataset, and 230 clinical data diagnosed with CAG from Hefei Second People’s Hospital from April 2018 to November 2024 as validation dataset to establish the predictive model. Both univariate and multivariate logistic regression analysis were employed to investigate the risk factors of CAG based on R software 4.4.1. After that, our predictive model was evaluated by nomogram, receiver operating characteristic (ROC) curve for discrimination of the predictive model, calibration curves, Hosmer-Lemeshow goodness of fit test for uniformity between the predicted and actual probabilities and decision curve analysis (DCA) curves for clinical validity.

**Results:**

Our multivariate logistic regression analysis revealed that depression disorder, drinking consumption, family history of digestive disorders, HP infection, pepsinogen I, pepsinogen II and gastrin 17 were the independent risk factors of our predictive model. A nomogram of CAG was established. The ROC curve revealed that our predictive model showed the best predictive efficacy with an AUC of 0.827 (95%CI = 0.784–0.870), with a specificity of 0.838 and sensitivity of 0.705 in training dataset, and an AUC of 0.970 (95%CI = 0.945–0.995), with a specificity of 0.881 and sensitivity of 0.950 in the validation dataset. Hosmer-Lemeshow goodness of fit test showed that our predictive model had a good fit for the training dataset (X-squared = 3.8293, df = 8, *p* = 0.8722) and validation dataset (X-squared = 8.9753, df = 8, *p* = 0.3444). Moreover, calibration and DCA curves demonstrated that our predictive model had a good fit, better net benefit and predictive efficiency in patients with CAG.

**Conclusion:**

Our predictive model demonstrated that depression disorder, drinking consumption, family history of digestive disorders, HP infection, pepsinogen I, pepsinogen II and gastrin 17 were the independent risk factors of CAG with high accuracy and good calibration.

## Introduction

Chronic atrophic gastritis (CAG) is a precancerous condition characterized by the loss of gastric glandular cells and their replacement by intestinal-type epithelium or fibrous tissue (1). Globally, its prevalence in 2023 remains significant, with estimates suggesting that 10–20% of adults over 50 years are affected (2), particularly in regions with high *Helicobacter pylori* (HP) infection rates, such as East Asia and Eastern Europe (2). CAG develops through persistent inflammation triggered by HP, autoimmune responses (e.g., anti-parietal cell antibodies), or environmental factors like smoking and high-salt diets (3). Over time, chronic inflammation leads to mucosal atrophy, intestinal metaplasia, and dysplasia, which are recognized as sequential steps toward gastric adenocarcinoma (4, 5). Several long-term follow-up researches revealed that less than 2% of CAG patients progress to gastric cancer annually, with intestinal metaplasia and dysplasia serving as critical histological markers for risk stratification (6–8).

Some key factors influencing CAG progression include the extent of intestinal metaplasia, dysplasia severity, and genetic predisposition. The intestinal metaplasia, classified into incomplete subtypes, is strongly associated with increased cancer risk due to aberrant differentiation of gastric stem cells (9). Some studies indicated that the ratio of progression from gastric dysplasia to invasive malignancy was declared to be approximately 3–12% in participants with low-grade dysplasia, and 10–69% in patients with high-grade dysplasia (10, 11). And several long lasting follow-up cohort studies demonstrated a significant relationship between gastric dysplasia and an elevated incidence of CAG or gastric cancer (6, 12). Familial clustering studies reveal that germline mutations in CDH1 (E-cadherin) and polymorphisms in IL-1β and TNF-*α* genes heighten susceptibility to CAG and gastric cancer (13, 14). Depression has also been increasingly recognized as a potential risk factor for CAG. Psychological stress and depression can disrupt the normal regulation of the neuroendocrine system, affecting the gastric mucosal microcirculation and the function of the immune system (15). Blood indicators can provide important clues for the diagnosis and assessment of CAG. For example, the levels of pepsinogen I and pepsinogen II in the blood are often used to evaluate the status of the gastric mucosa. A decrease in the ratio of pepsinogen I/pepsinogen II is associated with the atrophy of the gastric corpus mucosa (16). However, the relationship between subject characteristics and CAG still remains unclear. In our multi-center retrospective analysis, we examine‌d the clinical records with both social-demographic and clinicopathologic characteristics and chemical examination data in laboratory from patients with CAG to establish a predictive model. The aim of our study was to conduct our predictive model and assess the diagnostic efficacy of relevant indicators for the diagnosis of CAG in order to provide a reasonable basis for the diagnosis and treatment of CAG.

## Materials and methods

### Data collection

We retrospectively collected 539 patients who underwent CAG at the Second Affiliated Hospital of Anhui University of Chinese Medicine from September 2018 to December 2024 and considered them as training dataset. For the multi-center analysis, 230 participants diagnosed with CAG were also selected in Hefei Second People’s Hospital from April 2018 to November 2024, which were considered as validation dataset. Both training data and validation data were completed by gastroscopy procedure and pathological examination. Our multi-center retrospective analysis was approved by the Ethics Committee of both the Second Affiliated Hospital of Anhui University of Chinese Medicine and Hefei Second People’s Hospital. All participants in our multi-center retrospective study have signed the informed consent.

The inclusive criteria of CAG is as followed: (a) Endoscopic diagnosis, CAG shows a mixture of red and white mucous membranes, mainly white, with wrinkles flattening or even disappearing, and membrane blood vessels exposed, basic manifestations such as mucosal granules or nodules (17, 18). (b) Pathological diagnosis: Pathological biopsy shows intestinal metaplasia and (or) dysplasia, but multiple biopsies are needed to evaluate the extent and severity of atrophy (19). The exclusion criteria of CAG is as followed: (a) Individuals with CAG received drug treatment in the past; (b) Individuals who are disabled, including blindness, deafness, dumbness and so on; (c) Individuals who are not willing to join in our analysis; (d) Those who had incomplete or missing clinical data.

In our retrospective analysis, the logistic regression was utilized to construct our model, which is defined as one of the supervised learning. In order to prevent overfitting of our regression model, our dataset should be partitioned as training data and validation data (20), and the reasonable ratio is approximately 7: 3 (21). A predictive model of CAG is clinically valuable for identifying individuals at heightened risk of gastric mucosal degeneration and its progression to gastric cancer. Its applicability is particularly impactful in risk stratification and targeted screening, enabling precision-driven healthcare decisions, which the predictive model acts as a first-line tool to prioritize high-risk groups (e.g., HP-infected individuals, smokers, or those with familial gastric cancer history) for further evaluation.

### Data definition

#### Social-demographic characteristics

We recorded case history information of several social-demographic characteristics, including gender, age at diagnosis, education and marital status. Gender was categorized as male or female. The education level was defined as less than primary school, middle school or upper college. For marital status, it was defined as single status or married status.

#### Clinicopathologic characteristics

The following clinicopathologic characteristics were included in our present analysis: obesity (no, yes), hypertension (no, yes) or (Systolic blood pressure ≥ 140 or diastolic blood pressure ≥ 90), depression (no, yes), frailty (no, yes), drinking consumption (no, yes), smoking consumption (no, yes), diabetes (no, yes), family history (no, yes), dyslipidemia (no, yes) (cholesterol ≥ 6.19 or low density lipoprotein ≥ 4.14), HP infection (no, yes) or glucose (mmol/L), cholesterol (mmol/L), pepsinogen I (μg/L), pepsinogen II (μg/L), gastrin 17 (pmol/L), alpha fetoprotein (AFP) (ng/mL), carcinoembryonic antigen (CEA) (ng/mL), carbohydrate antigen 125 (CA125) (U/mL), carbohydrate antigen 199 (CA199) (U/mL). In this study, we used the Zung Depression Scale (SDS, self-rating depression scale) as a screening tool, and a standard score of greater than 50 was considered to be a possible depressive symptom. To improve the accuracy of diagnosis, we also invited specialists from the hospital’s psychological clinic to conduct clinical evaluations on patients with positive screening. Only when both the SDS screening results and the psychologist’s evaluation indicated depression was the final diagnosis of depressive disorder made.

### Model construction

The associations between CAG incidence and 25 potential risk factors or relevant disorders were investigated by R software. The chi-squared test was utilized to obtain the baseline information. For continuous variables followed the normal distribution, the baseline was presented as mean (standard deviation) and *p* value was calculated by *t* test. Clinical data followed the non-normal distribution, the baseline was presented as median (interquartile range) and p value was calculated by Mann–Whitney *U* test. Furthermore, categorical variables was showcased as number (N) or proportion (%). For large sample (*N* ≥ 40), the test method was chosen as chi-squared test, otherwise it is the Fisher’s exact test. After that, the univariate logistic regression analysis was used to analyze the recorded clinical data to control confounding factors. And the multivariate logistic regression analysis was further utilized to explore the association between independent risk factors of CAG.

### Validation process

The discrimination of our regression model was assessed with receiver operating characteristic (ROC) curve analysis, and a greater area under ROC (AUC) determined the better discriminative ability. For accuracy and clinical validity of our regression model, calibration curves and decision curve analysis (DCA) were utilized to evaluate.

### Details of statistical analysis

All the construction of clinical model and subsequent analysis are utilized with R version 4.4.1 (http://www.r-project.org, R Foundation for Statistical Computing). For baseline characteristics, we utilized “tableone” package, and obtained the table based on both “flextable” and “officer” packages. For establishing the linear regression model, “rms” package was used to plot nomograms for subsequent analysis. We plotted ROC curves and DCA curves based on “pROC” and “rmda” packages, respectively. A two-sided *p* value less than 0.05 was considered statistical significance.

## Results

### Participant baseline characteristics

In the current study, we finally obtained 152 patients with CAG, according to the aforementioned inclusive and exclusive criteria, of which 132 CAG patients from training cohort and 20 CAG patients from validation cohort. The mean age of training cohort was 60.87 ± 11.61, with slightly predominant number for female (*N* = 287, 53.2%). The majority of training population was married (*N* = 533, 98.9%) and dyslipidemia (*N* = 506, 93.9%). For daily habits and customs, the percentage ratio of individuals with drinking consumption (*N* = 174, 32.3%) and smoking consumption (*N* = 166, 30.8%) is close. The proportion of family history with CAG (*N* = 39, 7.2%) and HP infection (*N* = 51, 9.5%) is also similar. For relevant index of CAG in training cohort, the mean (standard deviation) of pepsinogen I, pepsinogen II and gastrin 17 are 120.30 (25.46), 11.03 (3.99) and 8.80 (3.69), respectively. Detailed baseline information of social-demographic characteristics and clinicopathologic characteristics of both training cohort and validation cohort is showed in Table 1.

**Table 1:** Baseline characteristics based on patients with non-CAG and CAG.

Subject characteristic	Training cohort (*N* = 539)		Validation cohort (*N* = 230)			
	Non-CAG	CAG	*p*-value	Non-CAG	CAG	*p*-value
Gender			0.118			0.151
Male	182 (44.7)	70 (53.0)		104 (49.5)	6 (30.0)	
Female	225 (55.3)	62 (47.0)		106 (50.5)	14 (70.0)	
Age	60.99 (12.13)	60.52 (9.87)	0.691	59.15 (12.29)	66.00 (10.34)	0.017
Education			0.513			0.851
Less than primary school	174 (42.8)	57 (43.2)		72 (34.3)	6 (30.0)	
Middle school	122 (30.0)	45 (34.1)		57 (27.1)	5 (25.0)	
Upper college	111 (27.3)	30 (22.7)		81 (38.6)	9 (45.0)	
Marital status			0.977			0.917
No	4 (1.0)	2 (1.5)		4 (1.9)	1 (5.0)	
Yes	403 (99.0)	130 (98.5)		206 (98.1)	19 (95.0)	
Obesity			0.662			0.891
No	239 (58.7)	74 (56.1)		166 (79.0)	15 (75.0)	
Yes	168 (41.3)	58 (43.9)		44 (21.0)	5 (25.0)	
Hypertension			<0.001			0.010
No	125 (30.7)	72 (54.5)		53 (25.2)	11 (55.0)	
Yes	282 (69.3)	60 (45.5)		157 (74.8)	9 (45.0)	
Depression			<0.001			0.455
No	83 (20.4)	59 (44.7)		53 (25.2)	3 (15.0)	
Yes	324 (79.6)	73 (55.3)		157 (74.8)	17 (85.0)	
Frailty			0.023			0.549
No	144 (35.4)	62 (47.0)		61 (29.0)	4 (20.0)	
Yes	263 (64.6)	70 (53.0)		149 (71.0)	16 (80.0)	
Drinking consumption			0.001			0.032
No	292 (71.7)	73 (55.3)		149 (71.0)	9 (45.0)	
Yes	115 (28.3)	59 (44.7)		61 (29.0)	11 (55.0)	
Smoking consumption			0.003			0.032
No	296 (72.7)	77 (58.3)		149 (71.0)	9 (45.0)	
Yes	111 (27.3)	55 (41.7)		61 (29.0)	11 (55.0)	
Diabetes			0.018			0.847
No	292 (71.7)	109 (82.6)		105 (50.0)	9 (45.0)	
Yes	115 (28.3)	23 (17.4)		105 (50.0)	11 (55.0)	
Family history			<0.001			<0.001
No	400 (98.3)	100 (75.8)		191 (91.0)	7 (35.0)	
Yes	7 (1.7)	32 (24.2)		19 (9.0)	13 (65.0)	
Dyslipidemia			0.808			1.000
No	381 (93.6)	125 (94.7)		202 (96.2)	19 (95.0)	
Yes	26 (6.4)	7 (5.3)		8 (3.8)	1 (5.0)	
HP infection			<0.001			<0.001
No	392 (96.3)	96 (72.7)		196 (93.3)	8 (40.0)	
Yes	15 (3.7)	36 (27.3)		14 (6.7)	12 (60.0)	
Glucose, mmol/L	5.16 (0.82)	5.59 (1.13)	<0.001	4.99 (0.66)	5.02 (0.72)	0.842
Cholesterol, mmol/L	4.39 (1.00)	4.46 (0.98)	0.500	4.24 (0.92)	4.53 (0.96)	0.187
Pepsinogen I, μg/L	124.44 (23.40)	107.52 (27.33)	<0.001	121.90 (23.03)	88.83 (29.75)	<0.001
Pepsinogen II, μg/L	11.35 (4.09)	10.05 (3.48)	0.001	11.92 (4.26)	6.73 (2.93)	<0.001
Gastrin17, pmol/L	9.28 (3.59)	7.35 (3.64)	<0.001	8.57 (3.63)	5.31 (3.68)	<0.001
AFP, ng/mL	6.81 (6.03)	7.55 (6.12)	0.222	9.52 (6.07)	10.41 (4.89)	0.525
CEA, ng/mL	2.21 (2.62)	2.24 (1.54)	0.900	2.70 (1.56)	2.40 (1.62)	0.413
CA125, U/mL	14.54 (15.52)	14.45 (10.38)	0.952	19.49 (9.04)	21.57 (8.57)	0.324
CA199, U/mL	18.20 (29.53)	22.40 (38.47)	0.190	19.20 (10.96)	13.63 (8.26)	0.028

AFP, alpha fetoprotein; CEA, carcinoembryonic antigen; CA125, carbohydrate antigen 125; CA199, carbohydrate antigen 199. Statistical significance, *p* < 0.05.

In order to present model development steps clearly, we calculated all characteristics considered with their unadjusted and adjusted odds ratios for characteristic selection strategy, and variance inflation factors (VIFs) for handling of multicollinearity as shown in Supplementary Tables S1–S4. Since the VIF values of the characteristics are all less than 10, there is no multicollinearity problem in the subsequent model construction.

### Univariate and multivariate analysis of CAG risk factors

In our univariate logistic regression analysis, a total of 12 potential risk factors of CAG in the training dataset showed statistically significant: hypertension, depression, frailty, drinking consumption, smoking consumption, diabetes, family history, HP infection, glucose, pepsinogen I, pepsinogen II and gastrin 17 (*p* < 0.05), as shown in Table 2. Hence, the variables with p < 0.05 were selected for the input of multivariate logistic regression analysis.

**Table 2:** The univariate analysis of common risk factors for CAG.

Subject characteristic	OR(95%CI)	*p*-value
Age	0.997 (0.980–1.014)	0.691
Education		
Middle school vs. Less than primary school	1.126 (0.713–1.772)	0.609
Upper college vs. Less than primary school	0.825 (0.495–1.355)	0.453
Marital status married vs. single	0.645 (0.124–4.692)	0.615
Obesity yes vs. no	1.115 (0.749–1.656)	0.590
HTN yes vs. no	0.369 (0.246–0.551)	<0.001
Depression yes vs. no	0.317 (0.208–0.482)	<0.001
Frailty yes vs. no	0.618 (0.415–0.921)	0.018
Drinking consumption yes vs. no	2.052 (1.367–3.078)	<0.001
Smoking consumption yes vs. no	1.905 (1.263–2.865)	0.002
Diabetes yes vs. no	0.536 (0.319–0.869)	0.014
Family history yes vs. no	18.286 (8.292–46.227)	<0.001
Dyslipidemia yes vs. no	0.821 (0.322–1.840)	0.652
HP infection yes vs. no	9.800 (5.252–19.137)	<0.001
Glucose, mmol/L	1.597 (1.300–1.985)	<0.001
Cholesterol, mmol/L	1.071 (0.877–1.304)	0.500
Pepsinogen I, μg/L	0.973 (0.965–0.981)	<0.001
Pepsinogen II, μg/L	0.920 (0.873–0.967)	0.001
Gastrin17, pmol/L	0.863 (0.814–0.912)	<0.001
AFP, ng/mL	1.020 (0.988–1.052)	0.224
CEA, ng/mL	1.005 (0.914–1.086)	0.900
CA125, U/mL	1.000 (0.984–1.013)	0.952
CA199, U/mL	1.003 (0.998–1.010)	0.226

AFP, alpha fetoprotein; CEA, carcinoembryonic antigen; CA125, carbohydrate antigen 125; CA199, carbohydrate antigen 199. Statistical significance, *p* < 0.05.

Multivariate logistic regression analysis further showed depression (OR = 0.856, 95%CI = 0.799–0.916), drinking consumption (OR = 1.076, 95%CI = 1.009–1.148), family history (OR = 1.584, 95%CI = 1.408–1.782), HP infection (OR = 1.469, 95%CI = 1.324–1.629), pepsinogen I (OR = 0.997, 95%CI = 0.995–0.998), pepsinogen II (OR = 0.987, 95%CI = 0.980–0.995) and gastrin17 (OR = 0.987, 95%CI = 0.979–0.995) are independent risk factors of CAG (*p* < 0.05), as presented in Table 3.

**Table 3:** The multivariate analysis of relevant risk factors for CAG.

Subject characteristic	*β*	sx	Walds	OR(95%CI)	*p*-value
Depression	−0.156	0.035	−4.469	0.856 (0.799–0.916)	<0.001
Drinking consumption	0.073	0.033	2.246	1.076 (1.009–1.148)	0.025
Family history	0.46	0.06	7.663	1.584 (1.408–1.782)	<0.001
HP infection	0.384	0.053	7.262	1.469 (1.324–1.629)	<0.001
Pepsinogen I	−0.003	0.001	−5.698	0.997 (0.995–0.998)	<0.001
Pepsinogen II	−0.013	0.004	−3.403	0.987 (0.980–0.995)	<0.001
Gastrin17	−0.013	0.004	−3.104	0.987 (0.979–0.995)	0.002

Statistical significance, *p* < 0.05.

**Figure 1:**
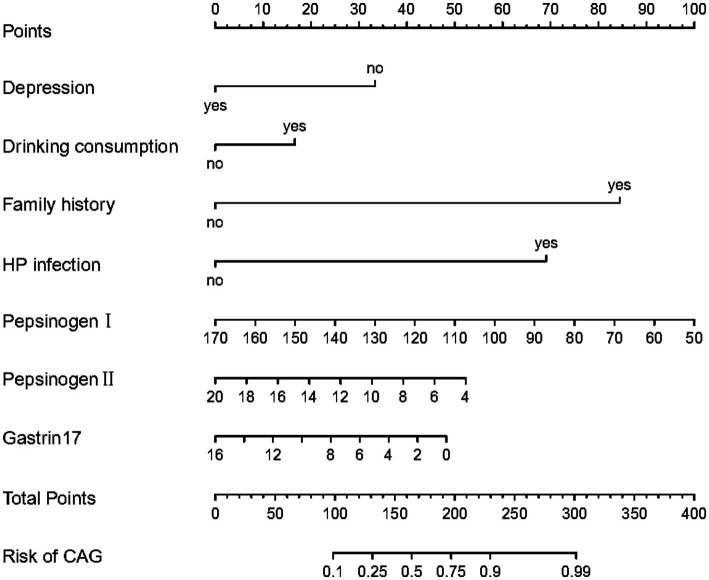
The nomogram for predictive model of CAG patients.

Meanwhile, the predictive model was showed as a nomogram, which constructed depression, drinking consumption, family history, HP infection, pepsinogen I, pepsinogen II and gastrin 17 based on the aforementioned risk factors or relevant disorders, as shown in Figure 1. Our nomogram offers a visual representation of the impact of each factor, helping doctors in conducting individualized risk evaluations in clinical practice. For example, if a patient with CAG had depression, had drinking consumption, had no family history, had no HP infection with pepsinogen I (120 μg/L), pepsinogen II (14 μg/L) and gastrin17 (12 pmol/L), then the patient corresponding scores would be about 33, 15, 0, 0, 41,18 and 13, respectively, with a total score of 120. This would revealed that the estimated probability of CAG patients is approximately 19%.

### Predictive model establishment

Based on the results of multivariate logistic regression analysis, the fitting equation of our predictive model is as followed:


$$\begin{array}{l} \text{Logit}(P)=-0.156+0.073x_{1}+0.46x_{2}+0.384x_{3}-\\ 0.003x_{4}-0.013x_{5}-0.013x_{6} \end{array}$$

where $x_{1}$ represents depression (No means 0, Yes means 1), $x_{2}$ represents drinking consumption (No means 0, Yes means 1), $x_{3}$ represents family history (No means 0, Yes means 1), $x_{4}$ represents HP infection (No means 0, Yes means 1), $x_{5}$ represents pepsinogen I value, $x_{6}$ represents pepsinogen II value, and the constant term of the formula (–0.156) means reference intercept.

### Validation and calibration of predictive model

**Figure 2:**
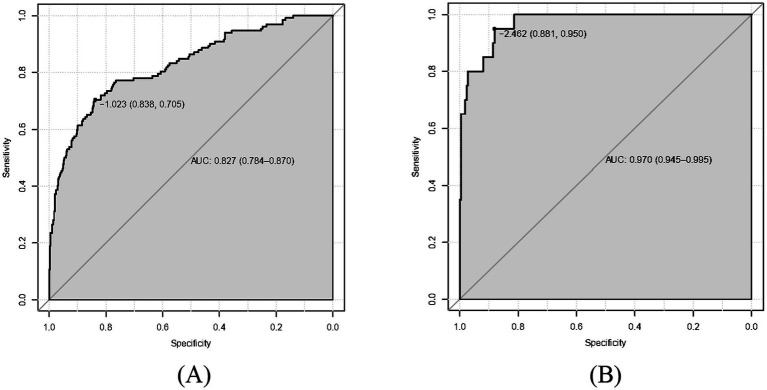
Receiver operating characteristic curve (ROC) of our predictive model. The area under curves (AUC) are 0.827 and 0.970 in training dataset and validation dataset, respectively, and our predictive model had a good accuracy. **(A)** Training dataset. **(B)** Validation dataset.

**Figure 3:**
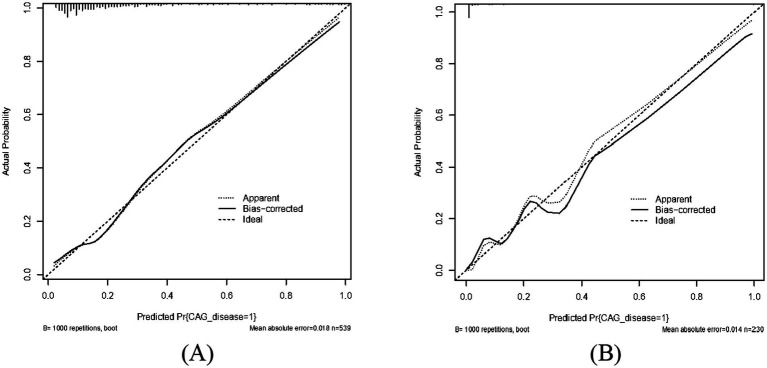
Calibrate curve of our predictive model. Both apparent curves and bias-corrected curves fit the ideal curve in training dataset and validation dataset with good fits for our predictive model. **(A)** Training dataset. **(B)** Validation dataset.

**Figure 4:**
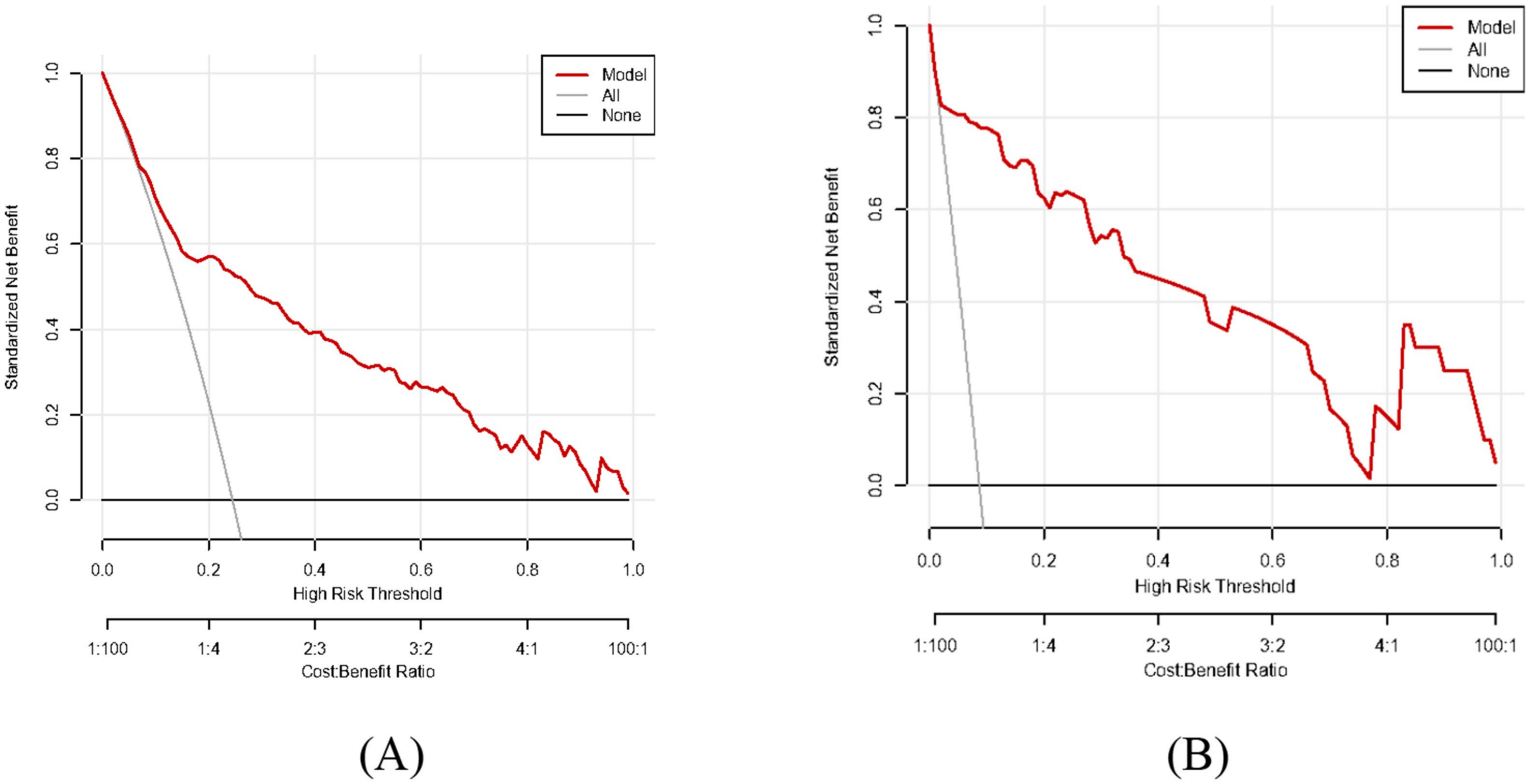
Decision curve analysis (DCA) of our predictive model. The model curve is located above all and none curve with good net benefit and accurate prediction. **(A)** Training dataset. **(B)** Validation dataset.

As shown in Figures 2A,B, the predictive model generated an AUC of 0.827 (95%CI = 0.784–0.870), with a specificity of 0.838 and sensitivity of 0.705 in the training dataset, and an AUC of 0.970 (95%CI = 0.945–0.995), with a specificity of 0.881 and sensitivity of 0.950 in the validation dataset. These results revealed that our predictive model had a good accuracy in individuals with CAG. Furthermore, the nomogram was assessed by a Hosmer and Lemeshow goodness of fit (GOF) test and calibration curve, and *p* value of Hosmer and Lemeshow GOF test greater than 0.05 represents the predictive model has a good degree of fit. The results showed that our model had a good fit for the training dataset (X-squared = 3.8293, df = 8, *p* = 0.8722) and validation dataset (X-squared = 8.9753, df = 8, *p* = 0.3444). For calibration curves, both training and validation dataset based on the multivariate logistic regression analysis are shown in Figures 3A,B. These results revealed that our predictive model had a good fit and predictive efficiency in patients with CAG. Finally, we utilized DCA curves to evaluate the clinical validity of our predictive model, as shown in Figures 4A,B. The net benefits of our model for the validation dataset from Hefei Second People’s Hospital were significantly higher than two extreme cases, revealing that the nomogram model had the better net benefit and accurate prediction.

## Discussion

CAG is a persistent inflammatory condition characterized by the loss of gastric glandular structures and thinning of the gastric mucosa (22). And this disease is often associated with HP infection, autoimmune mechanisms (e.g., autoimmune gastritis targeting parietal cells), or long-term exposure to irritants, encompassing alcohol consumption or certain medications (23). Several previous analysis have constructed clinical predictive models of CAG. A retrospective study constructed a predictive model for investigate the factors influencing the development of intestinal metaplasia or dysplasia in patients with CAG (24), and a retrospective research analyzed the risk of procession of gastric cancer in CAG patients, the association between lesion development and gastric mucosal background (25). In our multi-center retrospective analysis, a predictive model was established to assess the casual association with social-demographic and clinicopathologic characteristics of CAG patients, so as to provide a basis for both diagnosis and treatment of CAG patients in time. The findings of our multi-center retrospective analysis demonstrated that enormous independent factors influencing CAG included depression disorder, drinking consumption, family history of digestive disorders, HP infection, pepsinogen I, pepsinogen II and gastrin 17. In the relevant indicators, the clinical reference ranges of pepsinogen I is 70–165 μg/L, and a decrease in pepsinogen I levels indicates a decrease in gastric protease secretion ability, which may affect protein digestion function. More importantly, it is a sensitive indicator of gastric mucosal atrophy, and gastric mucosal atrophy (especially accompanied by intestinal metaplasia and dysplasia) is an important stage of gastric precancerous lesions. The clinical reference ranges of pepsinogen II is 3–15 μg/L, and elevated pepsinogen II indicates active damage or inflammation of the gastric mucosa. Finally, the clinical reference ranges of gastrin 17 is 1–15 pmol/L, and reduced secretion of gastrin 17 is an important indicator for evaluating gastric antral atrophy.

Our study revealed that the depression disorder was a predictor of CAG patients. The stress response activated by depression leads to an increase in the production of stress - related hormones like cortisol (26). Prolonged elevation of cortisol levels can damage the gastric mucosa, making it more vulnerable to the effects of gastric acid and harmful substances (27). As a result, it becomes more difficult for the damaged gastric mucosa to repair itself, accelerating the progression of CAG (28). Additionally, depression often leads to unhealthy lifestyle changes such as irregular eating habits and reduced physical activity, further aggravating the symptoms and condition of CAG (29). Moreover, alcohol consumption has a detrimental relationship with CAG. A study demonstrated that excessive amounts of alcohol consumption directly assaults the gastric mucosa (30). Furthermore, a finding demonstrated that alcohol intake is a strong irritant that can disrupt the protective mucus layer in the stomach. This mucus layer is vital as it shields the underlying tissue from the corrosive effects of gastric acid and the gastric mucosa becomes more exposed, making it easier for acid to erode the tissue and finally leading to CAG/gastric cancer (31). Besides, a family history of gastric cancer is significantly related to the occurrence and development of CAG, elevating the likelihood of developing CAG and the inclination for performing to gastric cancer (32).

Pepsinogen I, mainly secreted by the chief cells in the gastric fundus and body, is the precursor of pepsin, which is essential for protein digestion (33). In the early stage of CAG, as the gastric mucosa in the fundus and body starts to be damaged, the secretion of pepsinogen I decreases. A recent study indicated that a lower level of pepsinogen I is often an early biomarker indicating the onset of atrophy in these areas (34). Additionally, pepsinogen II, secreted not only by the chief cells in the fundus and body but also by pyloric glands and duodenal Brunner’s glands, shows a more complex change (35). With the progression of CAG, the damaged gastric mucosa leads to an increase in pepsinogen II levels in some cases (36). The ratio of pepsinogen I/pepsinogen II is often used as an important diagnostic indicator, as a decreasing pepsinogen I/pepsinogen II ratio is closely associated with the development and severity of CAG (37). Gastrin 17, a hormone secreted by G cells in the antrum of the stomach, is crucial for regulating gastric acid secretion (38). In CAG, especially when the antrum is affected, the production of gastrin 17 can be disrupted. Several studies revealed that a reduction in gastrin 17 levels may occur due to the atrophy of G cells, which impairs the normal acid - regulating mechanism in the stomach (39, 40). This can result in abnormal gastric acidity, further damaging the gastric mucosa and advancing to CAG.

To the best of our knowledge, HP infection is a major pathogen in the development of CAG. This bacterium can colonize the stomach mucosa, secrete urease and other virulence factors (41). The urease breaks down urea into ammonia, which damages the mucus layer protecting the gastric mucosa. The immune response triggered by HP infection causes chronic inflammation, gradually leading to the atrophy of gastric glands and the development of CAG (42, 43). Hence, the presence of HP significantly increases the risk of developing this chronic gastric disease and its related complications, and its eradication is often an important part of the treatment for CAG (44).

Numerous studies have also analyzed the CAG-related problems. Two meta-analysis and updated systematic reviews have provided an updated overview of the prevalence of CAG in the recent 10 years and its association with HP infection from 2010 to 2020 (45). In subgroup analysis, this study indicated that total risk ratio between HP infection and CAG risk is RR = 2.40 (95% CI: 2.16–2.67), and have higher risk during histological diagnosis (RR = 2.78, 2.49–3.12). In addition, the other research assessed the magnitude and nature of the association between CAG through histological and serological methods, and the incidence risk of upper gastrointestinal cancers from public database inception until August 10, 2023 (46). This study revealed that the incidence risk of CAG patients significantly increased by 4.12 times (OR = 4.12, 95% CI: 3.20–5.30), and the risk of CAG in histological diagnosis (OR = 4.23) is higher than that in serological diagnosis (OR = 3.88). Mechanism hypothesis is CAG may increase cancer risk through mechanisms such as HP infection, chronic inflammation, and reduced gastric acid secretion. These novel studies have confirmed the effectiveness and reliability of our clinical predictive model, which demonstrated that there is a correlation between the CAG influencing factors of other studies and the influencing factors of our clinical prediction model.

In patients with CAG, serum levels of pepsinogen and gastrin-17 exhibit characteristic changes reflecting gastric mucosal damage and functional impairment. Pepsinogen I is mainly secreted by the chief cells of the gastric corpus/fundus. As CAG develops and the oxyntic (acid-producing) mucosa undergoes atrophy, there is a significant reduction in the number of chief cells (47). Consequently, serum pepsinogen I levels will be very low. The pepsinogen I/pepsinogen II ratio—where pepsinogen II is more diffusely produced in the stomach, including the antrum—is an even more sensitive indicator. This ratio is significantly decreased in CAG, particularly when corpus atrophy is present. Low pepsinogen I and low pepsinogen I/pepsinogen II ratio are strong biochemical markers of corpus mucosal atrophy (48). Gastrin 17 is predominantly secreted by G-cells located in the gastric antrum. Its primary physiological function involves stimulating gastric acid secretion from the parietal cells lining the corpus. In cases of CAG involving the corpus, there is functional loss of parietal cells and resultant hypo-or achlorhydria (49). Acid deficiency precludes the possibility of normal inhibitory feedback on the G-cells. For this reason, fasting serum gastrin 17 is often markedly elevated in patients with CAG, especially those with corpus-predominant atrophy. High gastrin 17 is an expression of an attempt from the body to compensate for low acid production; in this case, the damaged or lost target cells are the parietal cells (50). The hallmark serological pattern for CAG is low pepsinogen I, low pepsinogen I/pepsinogen II ratio, and elevated gastrin 17. In this combination, a noninvasive marker for gastric mucosal atrophy, especially that which occurs in the corpus, and functional hypochlorhydria provides assistance in diagnosing and stratifying risk, for example, within gastric cancer of CAG.

Furthermore, there are some limitations in our multi-center retrospective analysis. First of all, the record size of our analysis is relatively small, and this limits the generalizability of our study. Meanwhile, the diagnoses of depression, frailty, drinking consumption, smoking consumption and family history were based on self-reported dataset from medical record data in two hospitals, which may cause information bias due to the cognitive errors of patients, and depression diagnosed or recorded has potential misclassification due to self-reporting. In addition, the exclusion criteria eliminate individuals unwilling to participate or those with disabilities. This may limit representativeness, which may affect the model’s applicability and validity. Subsequently, our analysis only performed internal validation. While internal validation is performed using a separate cohort from a second hospital, this still constitutes temporal or geographical internal validation without generalizability. Future external validations will be implemented across broader populations and different hospitals for generalizability of the model. The transportability of nomogram in additional CAG patients is supposed to be further validated. Meanwhile, the characteristics of gastric mucosa atrophy details, including Kimura-Takemoto classification, intestinal metaplasia and dysplasia were not recorded and ranked. These important features should be evaluated in subsequent analysis. Additionally, the lack of Kimura-Takemoto classification or histological severity scoring reduces clinical insight, and our method had no comparison with existing models for CAG detection. Finally, our predictive model only utilized the logistic regression method. More machine learning methods, including Random Forest, XGBoost, SVM, can be employed for performance comparison and potential improvement over logistic regression in the future with cross-validation or bootstrapping to avoid overfitting, and nomogram of multivariate logistic regression analysis can be enhanced for clinical utility by an online calculator or app in the further analysis.

## Conclusion

In our multi-center retrospective analysis, we found an association between several independent factors (depression disorder, drinking consumption, family history of digestive disorders, HP infection, pepsinogen I, pepsinogen II and gastrin 17) and CAG and established a predictive model to evaluate the clinical diagnosis of CAG.

## Data Availability

The raw data supporting the conclusions of this article will be made available by the authors, without undue reservation.
